# Rationale for the design of 3D-printable bioresorbable tissue-engineering chambers to promote the growth of adipose tissue

**DOI:** 10.1038/s41598-020-68776-8

**Published:** 2020-07-16

**Authors:** Pierre Faglin, Marion Gradwohl, César Depoortere, Nicolas Germain, Anne-Sophie Drucbert, Stéphanie Brun, Claire Nahon, Salim Dekiouk, Alexandre Rech, Nathalie Azaroual, Patrice Maboudou, Julien Payen, Pierre-Marie Danzé, Pierre Guerreschi, Philippe Marchetti

**Affiliations:** 1 Hôpital Salengro –Service de Chirurgie Plastique, CHU Lille, 59000 Lille, France; 2 Centre de Biologie Pathologie - Banque de Tissus, CHU Lille, 59000 Lille Cedex, France; 3 CHU Lille, IRCL, CNRS, Inserm UMR9020-UMR-S 1277 Canther, Univ. Lille, 59000 Lille, France; 4 Inserm, CHU Lille, U 1008, Univ. Lille, 59000 Lille, France; 5 Lattice Medical, 70 Rue du Docteur Yersin, 59120 Loos, France; 6 Plateau RMN, Faculté de Pharmacie, EA 7365 GRITA, 59000 Lille, France; 7 CHU Lille, ULR 7365 - GRITA - Groupe de Recherche Sur Les Formes Injectables Et Les Technologies Associées, Univ. Lille, 59000 Lille, France

**Keywords:** Translational research, Breast cancer

## Abstract

Tissue engineering chambers (TECs) bring great hope in regenerative medicine as they allow the growth of adipose tissue for soft tissue reconstruction. To date, a wide range of TEC prototypes are available with different conceptions and volumes. Here, we addressed the influence of TEC design on fat flap growth in vivo as well as the possibility of using bioresorbable polymers for optimum TEC conception. In rats, adipose tissue growth is quicker under perforated TEC printed in polylactic acid than non-perforated ones (growth difference 3 to 5 times greater within 90 days). Histological analysis reveals the presence of viable adipocytes under a moderate (less than 15% of the flap volume) fibrous capsule infiltrated with CD68^+^ inflammatory cells. CD31-positive vascular cells are more abundant at the peripheral zone than in the central part of the fat flap. Cells in the TEC exhibit a specific metabolic profile of functional adipocytes identified by ^1^H-NMR. Regardless of the percentage of TEC porosity, the presence of a flat base allowed the growth of a larger fat volume (*p* < 0.05) as evidenced by MRI images. In pigs, bioresorbable TEC in poly[1,4-dioxane-2,5-dione] (polyglycolic acid) PURASORB PGS allows fat flap growth up to 75 000 mm^3^ at day 90, (corresponding to more than a 140% volume increase) while at the same time the TEC is largely resorbed. No systemic inflammatory response was observed. Histologically, the expansion of adipose tissue resulted mainly from an increase in the number of adipocytes rather than cell hypertrophy. Adipose tissue is surrounded by perfused blood vessels and encased in a thin fibrous connective tissue containing patches of CD163^+^ inflammatory cells. Our large preclinical evaluation defined the appropriate design for 3D-printable bioresorbable TECs and thus opens perspectives for further clinical applications.

## Introduction

Reconstruction of adipose tissue defects after surgery (e.g. mastectomy) or trauma remains a major surgical challenge. Numerous tissue-engineering techniques were attempted experimentally. It includes repair methods such as autologous or heterologous fat grafting or also transfer of vascularized adipose tissue from a donor site, *also called a* fat flap. The limitation of this latter method is that the fat flap requires a sufficient amount of available fat in the donor site, which often leads to deformity at the donor site. Moreover, donor flap sites may not always be available. In order to limit these drawbacks, the fat flap method can be greatly improved when the fat flap is implanted within a tissue-engineering chamber (TEC). A TEC is a surgical device shaped as a hollow dome in which a small volume of fat flap with an independent vascular pedicle is inserted (for recent review^1^).

The in vivo TEC creates an uncollapsible space that allows the body’s own regenerative mechanisms to increase the volume of fat flaps, without added factors, cells or matrices, by stimulating adipose-derived stem cells differentiation and the proliferation of adipose precursor cells^2^. In comparison to the fat flap method, adding a relatively simplistic device such at the TEC lowers the amount of fat tissue harvested and therefore defects at the donor site. A TEC coordinates all the complex mechanisms that promote adipose tissue generation. The TEC implantation triggers surgical trauma and a foreign body reaction resulting in an acute sterile inflammation, which mimics the wound healing process. This early inflammatory stage (within 15 days post implantation)^2^ corresponds to a transient response of the body against the TEC and is characterized by an infiltration of macrophages and stem cells as well as local release of inflammatory and angiogenic factors. Soluble factors subsequently enhance angiogenesis, extracellular matrix remodeling, and eventually promote adipogenesis and adipose maturation^2^. Vascularization is key to developing large fat tissue facilitating long-term viability and function of the neo-tissue. It has been suggested that every adipocyte possess at least one supportive capillary^3^, which facilitates oxygen, nutrient, and waste exchange. Inflammation directly correlates with the TEC’s angiogenic response. TECs also promote angiogenic signals in response to gradual hypoxic conditions temporarily created by the chamber’s implantation^4^. There is a strong synergy between angiogenesis and adipogenesis responsible for the rapid fat flap growth under the TEC. Vascular endothelial cells per se*,* support the preadipocyte proliferation and differentiation partly via cell–cell interaction^5^ or through the secretion of extracellular matrix components^6^. Moreover, adipocyte stem cells, the main cell population that contributes to adipogenesis, are considered as stem cells of vascular origin and are located at proximity of blood vessels^7^. Conversely, mature adipocytes sustain angiogenesis through the secretion of multiple soluble factors (for review^3^). Interestingly, it has been demonstrated that macrophages are the cornerstone of both neo-angiogenesis and neo-adipogenesis in the TEC since their pharmacological depletion impedes new vessel formation and therefore adipose tissue development^8^. These intertwined effects contribute to the growing and maintaining of well-vascularized, viable, functional and mature adipose tissue under the TEC.

Besides angiogenesis, the chamber also creates a protected space for tissue growth that changes the mechanical forces on the fat flap. It has been evidenced that, unlike other tissues like muscles, adipogenesis is impeded by mechanical compression^9^. TEC creates a space that diminishes the mechanical tension of surrounding tissues on the fat flap thus promoting mitogenic stimuli to adipocyte lineage cells.

As a consequence of these complementary effects, the TEC acts as *a bona fide* bioreactor promoting an in vivo fivefold increase of the fat flap volume within several months^10^. This TEC technology has been experimentally used in a wide range of animal models including mice^11^, rats^4,12,13^, rabbits^14^ and pigs^10^. More recently, Morrison and al demonstrated the surgical feasibility and safety of the TEC for breast reconstruction in a first-in-human trial^15^. In fact, several groups have demonstrated the feasibility of the TEC device in the generation of vascularized, stable, mature and viable adipose tissue to repair body defects. One substantial limitation of the TEC method is its difficulty in producing adequate amounts of adipose tissue for clinical application. Indeed, due to the long-term persistence of exogeneous TEC in vivo, the initial acute inflammatory response can evolve towards the development of chronic local inflammation that leads to the development of a fibrous capsule on the fat flap surface. The formation of a thick contractile capsule further limits the expansion of the adipose tissue reducing the maximum final volume within the TEC and acting as a structural and biological barrier that prevents vascular access, oxygen and nutrient diffusion to the fat flap.

During the past few years, a wide variety of TEC prototypes, all in different shapes, were created with different exogeneous materials that persist long-term in vivo such as polycarbonate acrylic^16,17^ and polypropylene^18^.

Now, with the goal of overriding these limitations, studies should focus on improving these TEC devices in order to use them in clinical applications. Firstly, we decided to identify TEC criteria that are crucial to tissue growth and therefore define the optimum TEC device. We produced several TEC prototypes in polylactic acid with 3D printing technology enabling easily the conception of TEC devices with complex structures. Secondly, we used the bioresorbable polymer poly[1,4-dioxane-2,5-dione] (polyglycolic acid (PGA) PURASORB PGS) to produce short-lived TECs. We chose this GMP grade polymer because it is primarily used for sutures and is suitable for fused deposing modeling 3D printer. Based on these above findings, the main goal of our work was to improve these chambers for adipose tissue generation in addressing two complementary issues: the importance of TEC design and the possibility to use bioresorbable polymers for optimum chamber conception.

## Results

### Material characterization

**Figure 1:**
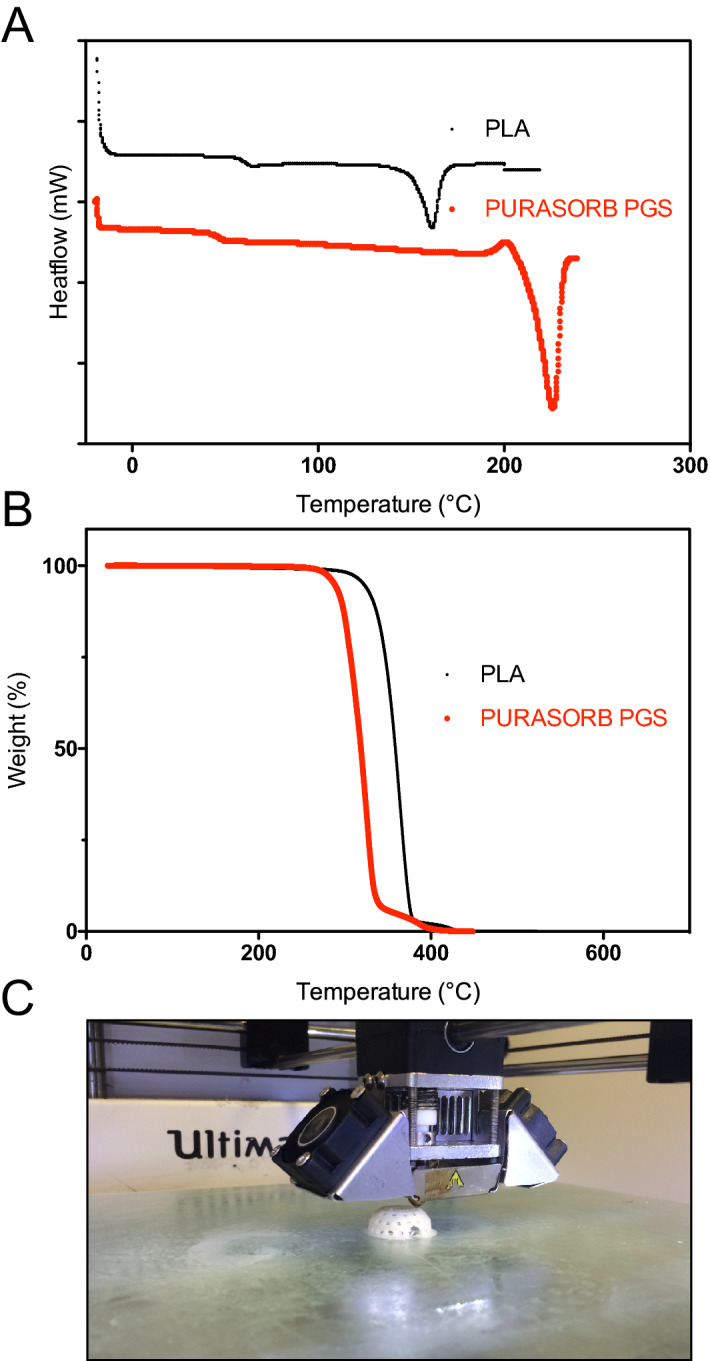
Material characterization and construction of TEC model (**a**) DSC curves of non-printed monofilaments of PLA (black) and PURASORB PGS (red) at a heating rate of 10 °C/min; (**b**) TGA curves of PLA (black) and PURASORB PGS (red); (**c**) Representative photomicrograph of TEC sample 3D-printed with PLA material for in vivo experiments.

Material characterization of PURASORB PGS and PLA by differential scanning calorimetry (DSC) (Fig. 1a) and thermogravimetric analysis (TGA) (Fig. 1b) was used to determine the optimal temperature necessary for the 3D printing of the bioresorbable polymers. Results of the DSC and TGA analyses are summarized in Table 1. PLA demonstrated glass transition temperature at 62 °C followed by one endothermic peak at 155 °C. whereas for PURASORB PGS Tg occurred at 45 °C and had a melting point at 155 °C (Fig. 1a). Both materials were semi-crystalline, but the DSC curves showed higher crystallinity rate for PURASORB PGS (Fig. 1a). The 5% weight loss temperatures (T5%), determined by TGA (Fig. 1b) were above the printing temperature for both polymers. Nevertheless, only PURASORB PGS displayed small gap between T5% and the melting point corresponding to early degradation of the material due to the impact of manufacturing processes. Figure 1c depicts the PLA 3D-printed TEC samples prepared for in vivo experiments.

**Table 1:** Properties of PLA and PGA.

Material	Tg (°C)	Tm (°C)	Td5% (°C)
PLA	62	155	308
PGA	45	226	288

### The importance of multi-perforated TEC

**Figure 2:**
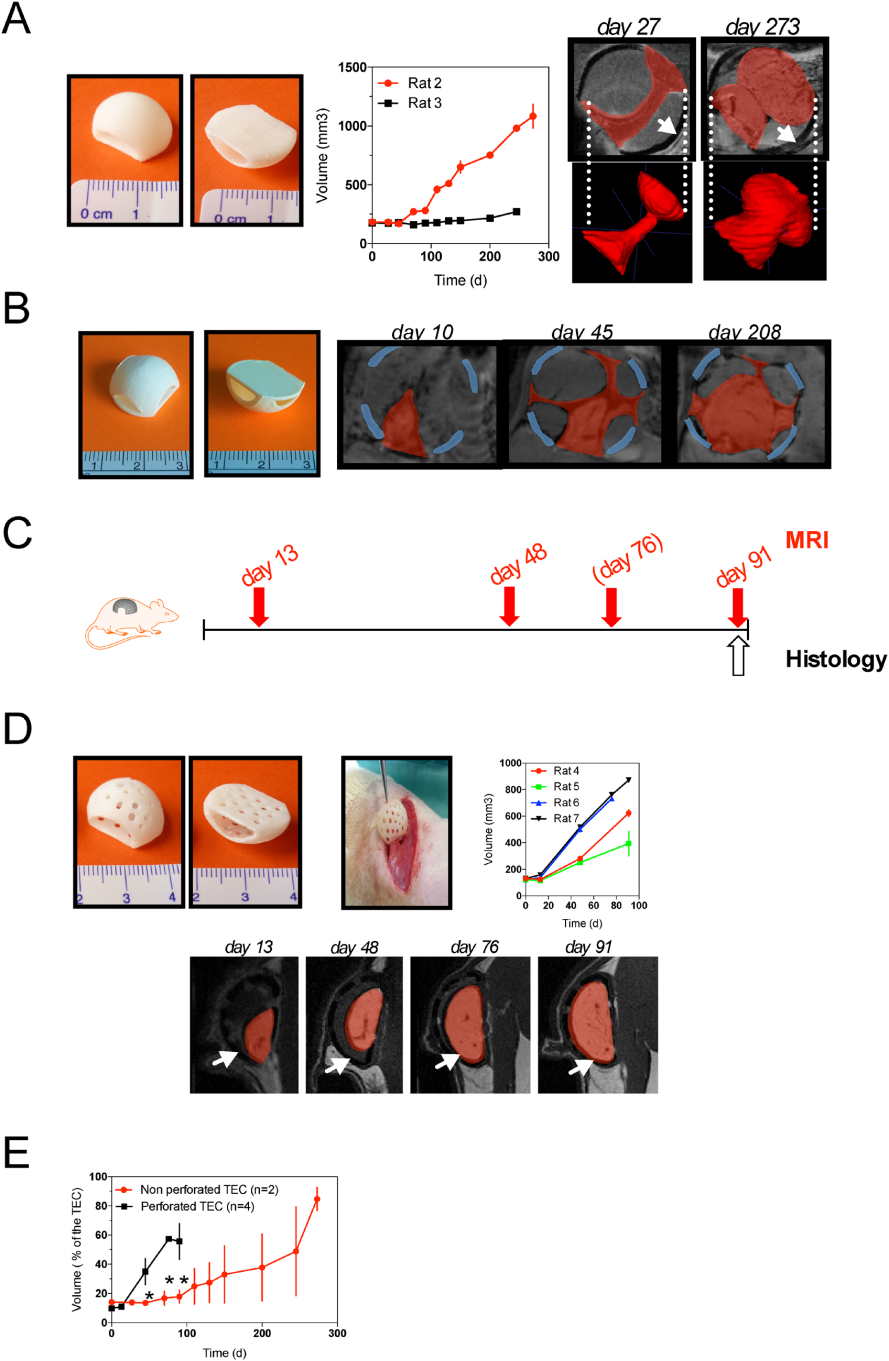
Influence of TEC porosity on fat flap growth (**a**) *(left)* The dome-shaped, non-perforated chamber design with polylactic acid (pictures from above and below) *(middle)* MRI Quantitative analysis of in vivo growth kinetics of adipose tissue within non-perforated TEC. Volume is determined from MRI images. Results are means ± SD of replicate measurements by 3 different operators in 2 rats (rat 2 and 3); *(right)* Magnetic resonance imaging scans and 3D reconstruction of adipose tissue within non-perforated TEC at day 27 and 273 after implantation. The size increased of the Fat Flap (red) can clearly be seen. (White arrows indicate the chamber); (**b**) Diagram showing the configuration of the TEC containing 4 holes; Magnetic resonance imaging scans (Multiplanar reconstruction) of adipose tissue within the 4-hole TEC at days 10, 45 and 208 after implantation. Adipose tissue (red) has heterogeneously increased size with the fastest growth located in front of the holes. (Blue lines indicate the chamber); (**c**) Experimental design: schematic depicting follow-up of rats implanted with multiperforated TEC (TEC 3 see Table 2) by MRI and histological evaluations at the time points indicated (MRI were performed at day 76 only for rats 6 and 7 (**d**) *(left)* The dome- shaped, perforated chamber design with polylactic acid (pictures from above and below) and its insertion in rat; *(middle)* MRI quantitative analysis of in vivo growth kinetics of adipose tissue within the perforated TEC. Results are expressed as means ± SD of replicate measurements by 3 different operators in 4 separated rats (rat 4, 5, 6 and 7) *(left)* Magnetic resonance imaging in sagittal views of adipose tissue within the perforated TEC at day 13, 48, 76, 91 after implantation. The increased size of the Fat Flap (red) can clearly be seen. (White arrows indicate the limit of the chamber); (**e**) Comparison of in vivo growth kinetics of adipose tissue within non-perforated (n = 2) and perforated TECs (n = 4). Results are expressed as means ± SD of percentages of the theoretical volume within the TEC.

**Figure 3:**
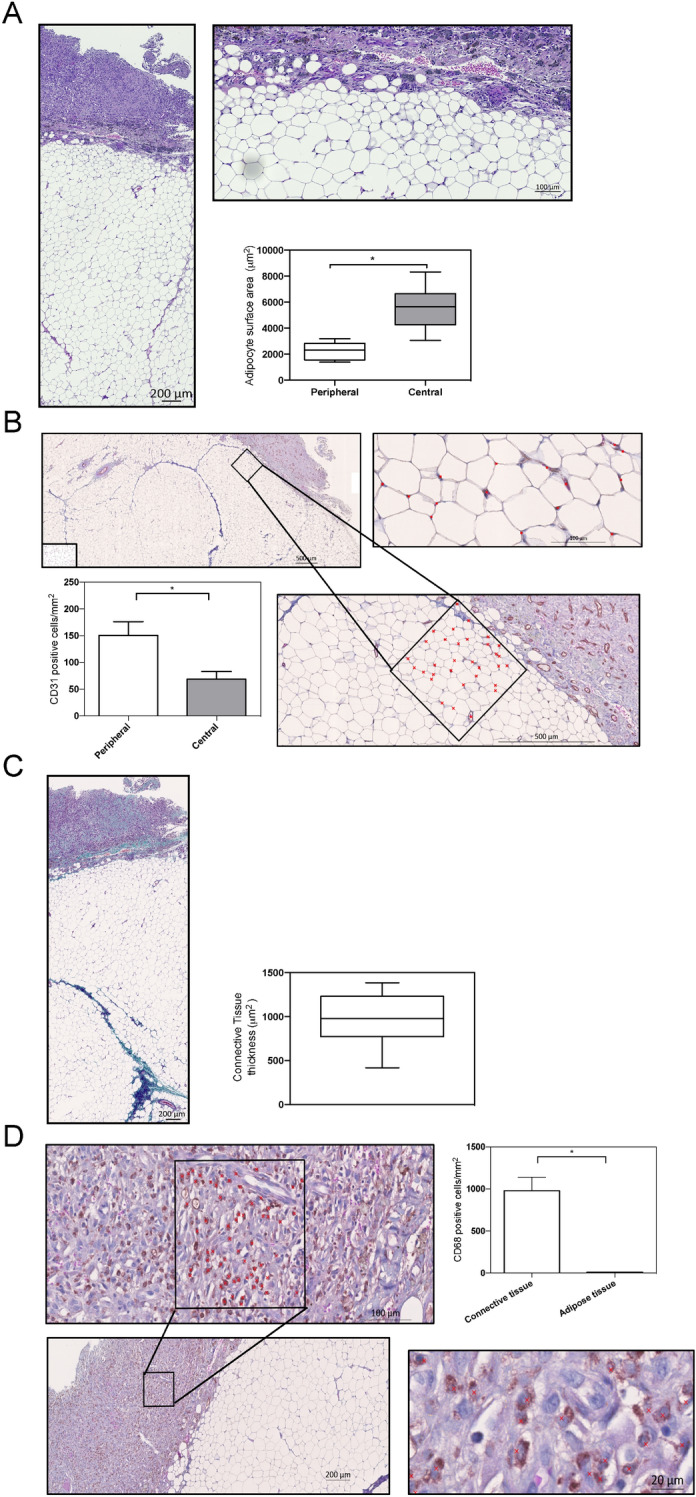
Histomorphometric analyses of fat flap under the multi-perforated TEC (**a**) Representative image of HE staining of new adipose tissue growing within the perforated TEC surrounded by connective tissue (*left*). Higher magnification view of a representative area under the connective tissue is shown on the *upper right*. *Lower right* Adipocyte surface area in the peripheral and central zone of the flap. Data are presented as Whiskers bar graphs (median 5–95 percentile). * p < 0.05; (**b**) Representative images of anti-CD31-stained sections of the chamber tissue. Higher magnification views of a representative area under the connective tissue are shown on the right part. (Red asterisks show CD31^+^ stained blood vessels). *Lower left*. Number of CD31^+^ cells in the peripheral and central zones of the flap. Data are mean + /SD * p < 0.05; (**c**) Representative image of Masson’s Trichrome stained tissue. Blue areas indicate collagen fibers deposition*.* (*right*) Note that adipose tissue components with septa were observed. Thickness of the connective tissue around the fat flap was measured. Data are presented as Whiskers bar graphs (median 5–95 percentile); (**d**) Representative images of anti-CD68-stained cells in the fat flap. Higher magnification views of a representative area in the connective tissue are shown. (Red asterisks show CD68 + stained macrophages). *Lower left*. Number of CD68^+^ cells in the connective and adipose tissues under the TEC. Data are mean + /SD * p < 0.05;

**Figure 4:**
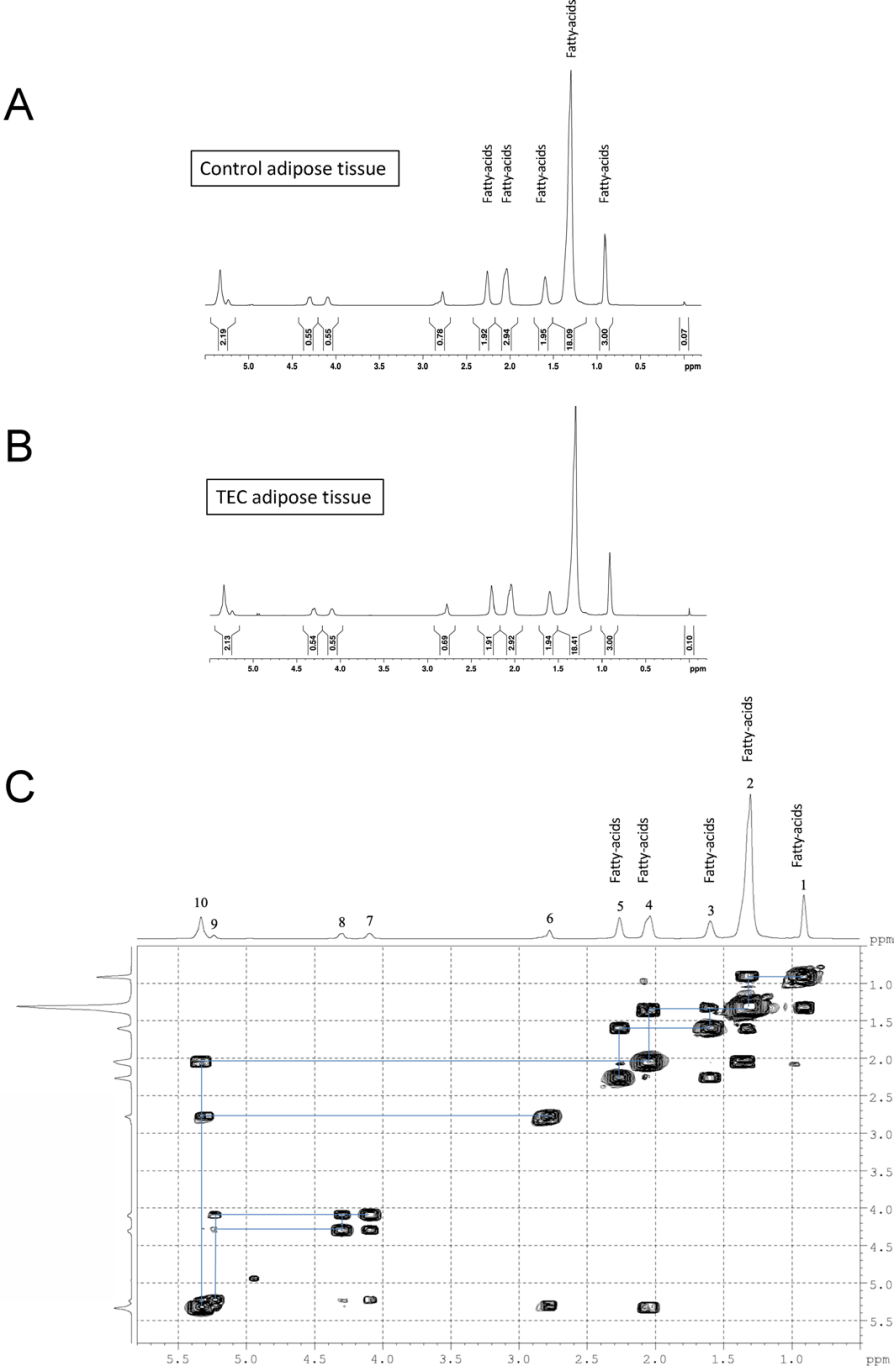
^1^H-NMR analysis of the metabolite profile of newly-generated adipose tissue in the TEC (**a**, **b**) Typical high-resolution 1D 1H-NMR spectrum of an extract of rat adipose tissue generated in the TEC (**b**) or of white adipose tissue serving as control (**a**, **c**) 2D COSY spectrum recorded from the adipose tissue in the TEC.

**Figure 5:**
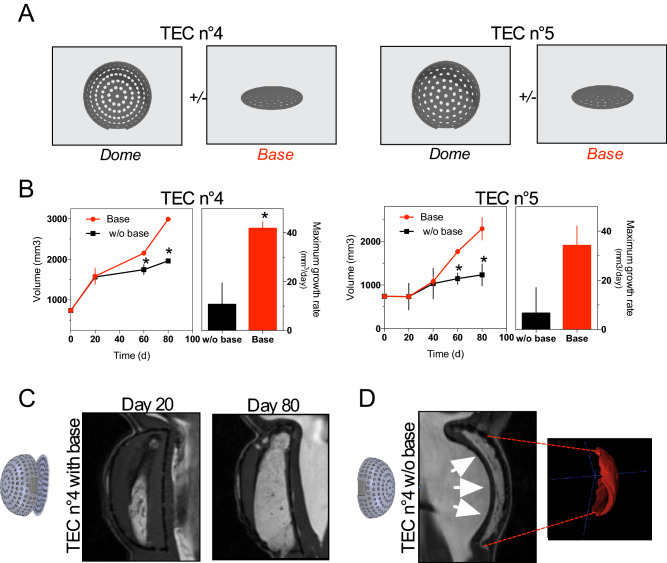
Influence of a TEC base on fat flap growth (**a**) Diagram showing the description of TEC4 and TEC5. The two tissue-engineering chambers (TEC 4 and TEC 5) differ by their dome porosity but have an identical flat base; (**b**) Growth curves of fat flap in the presence of TEC4 (left panel) or TEC5 (right panel) with (red line) or without a base (black line). Alternatively, maximum growth rates are calculated. Volume is determined on MRI images by three independent examiners. Values are expressed as mean ± SD (n = 3). * p < 0.05; (**c**) Representative longitudinal Magnetic Resonance Imaging (MRI) data of TEC4 with a base at day 20 *(left)* and day 80 *(right)* after implantation; (**d**) Representative longitudinal MRI data (left) and 3D modeling of fat flap (right) under TEC4 without a base at day 60 after implantation. White arrows show host tissues that fill the chamber impeding growth of the fat flap.

All 12 rats (Figs. 2 to 5) well tolerated the surgical procedure. No clinical signs of infection, or wound opening were observed at the first week postoperatively. We first analyzed the volume of fat flap for more than 200 days by iterative MR images in two rats (rat 2 and 3) implanted with non-perforated TEC (TEC 1, see Table 2). As seen in Fig. 1a, the volume of the fat flap did not change significantly during the time course in rat 3. Conversely, in rat 2, after a 100-day lag-period, the volume of newly-formed tissues had significantly increased at 150 days achieving more than fivefold its initial volume at day 273. Morphologically, MRI images revealed heterogeneous locations of newly growth tissue. The most important tissue growth was observed in front of the holes dedicated to the pedicle vessels (Fig. 2a,b). The heterogeneous aspect was also found for the tissue growing under a 4-holes TEC device (TEC 2, Table 2) where newly-formed tissues adopted a cross-like appearance over the study period (Fig. 2b).

**Table 2:** Characteristics of tissue engineering chambers (TEC) used in this study.

	TEC 1	TEC 2	TEC 3	TEC 4	TEC 5	TEC 6
Animal model (Figure in paper)	Rat (Fig. 2)	Rat (Fig. 2)	Rat (Figs. 2, 3, 4 )	Rat (Fig. 5)	Rat (Fig. 5)	Pig (Figs. 6, 7, 8)
Polymer	PLA (Chromatik)	PLA (Chromatik)	PLA (Chromatik)	PLA (Chromatik)	PLA (Chromatik)	PGA (Purasorb PGS Corbion)
Bioresorbable	No	No	No	No	No	Yes
3D Printer	Untimaker2 (Ultimaker, The Netherlands)	Ultimaker2 (Ultimaker, The Netherlands)	Ultimaker2 (Ultimaker, The Netherlands)	DiscoEasy200 (3D Dagoma, France)	DiscoEasy200 (3D Dagoma, France)	Ultimaker2 (Ultimaker, The Netherlands)
**Dome characteristics**						
Hole(s) on side(s) for insertion of vessels	2	4	2	1	1	1
Theorical volume of TEC (mm^3^)	1 279	1 279	1 279	5 999	5 999	114 000
Thickness (mm)	1	1	1	0.6	0.6	2
Diameter (mm)				30	30	80
Dome perforated	No	No	Yes	Yes	Yes	Yes
Porosity (%)	No	No	nd	28	19	19
**Pores**						
Number			27	210	148	148
Diameter (mm)			2	1.5	1.5	4
Distribution			Homogeneous	Homogeneous	Homogeneous	Homogeneous
**Base characteristics**						
Removable base	No	No	No	Yes	Yes	Yes
Thickness (mm)	1	1	1	1	1	0.5
Dimension (mm)			20 × 15	Diameter: 30	Diameter: 30	Diameter :80
Base perforated			Yes	Yes	Yes	Yes
Porosity (%)			14	21.5	21.5	21.5
**Pores**						
Number			13	86	86	86
Diameter (mm)			2	1.5	1.5	4
Distribution			Homogeneous	Homogeneous	Homogeneous	Homogeneous

Afterwards, we studied the growth of fat flap under multi-perforated TEC in 4 rats (TEC 3, Fig. 2c and 2d and see description in Table 2). Although the growth speed was different between rats (Fig. 2d), we observed in all of them a significant growth of the fat flap starting at day 13 post-implantation without the lag-period observed in non-perforated TEC conditions (Fig. 2e). Moreover, the morphology of these newly formed tissues was homogenous over the study period. In comparison, the difference in speed growth between the closed TEC and perforated TEC conditions was evident (Fig. 2e). Thus, we identified TEC porosity as a crucial factor to promote optimal growth of fat tissue.

HE staining of the samples harvested at 91 days was used to visualize the adipose tissue architecture under the multi-perforated TEC. As shown in Fig. 3a, the newly generated adipose tissue exhibited a mature architecture with fat cells organized into regular lobules that supported disseminated new vessels (Fig. 3a and b). The number of CD31^+^ vessels was significantly higher in the periphery of the engineered adipose tissue (Fig. 3b). The outermost layer of adipose tissue displayed a large number of small adipocytes (Fig. 3a) that interacted with CD31^+^ endothelial cells (Fig. 3b) revealing the tight association of adipogenesis with angiogenesis. HE staining also allowed to evaluate the thickness of the connective tissue surrounding the flap that represented around 13% of the flap (Fig. 3a and c). Masson’s trichrome staining (Fig. 3c) revealed the collagen deposition in the connective tissue with around 22.5% areas of collagen staining (blue). This flap capsule also contained CD68-positive macrophages quite rarely found in the newly formed adipose tissue (Fig. 3d). Overall, histomorphometric studies demonstrated that under the multi-perforated TEC the newly-formed tissue was predominantly composed of mature adipocytes and presented with disseminated blood vessels. Expanded adipose tissue was encapsulated by a thin connective tissue containing collagen fibers and macrophages.

Next, we performed the ex vivo ^1^H–NMR spectroscopy analysis of the adipose tissue formed in the TEC (Fig. 4). Analysis of the generated adipose tissue revealed comparable spectrum to that observed in the rat’s white adipose tissue (Fig. 4a and b), which represents the metabolic signature of functional mature adipose tissues^19,20^. This result was confirmed by the 2D COSY analysis of TEC-induced adipose tissue (Fig. 4c) that highlighted specific chemical shifts (Table 3) previously attributed to the spin system of fatty acids^20^. Thus, 1H-NMR analyses of fat tissue generated in the TEC showed a metabolite profile characteristic of fully functional adipose tissue.

**Table 3:** The resonance assignments, chemical shifts recorded from the adipose tissue in the TEC.

	Functional group	Chemical shift
1	CH_3_	0.89 ppm
2	–(CH_2_)_n_–CH_3_	1.30 ppm
3	–CH_2_–CH_2_–CO–	1.58 ppm
4	–CH_2_–CH=CH–	2.02 ppm
5	–CH_2_–CO–	2.25 ppm
6	= CH–CH_2_–CH=	2.78 ppm
7	–CO–O–CH–CH_2_–O–CO–C	4.1 ppm
8	–CO–O–CH–CH_2_–O–CO–C	4.3 ppm
9	–CO–O–CH(CH_2_–O–CO–C–)_2_	5.24 ppm
10	–CH=CH–	5.33 ppm

### The importance of the TEC base

To determine whether the presence of a flat base is important for the TEC design, we compared the growth of fat tissue under TEC with and without a flat base. We repeated the experiment with two multi-perforated TEC models (TEC 4 and TEC 5) bearing different porosity specificity (see Table 2) in the presence or absence of a flat base (Fig. 5a). Regardless of the presence of a base, under the TEC, the volume of tissue flaps increased over time (Fig. 5b and c). However, flaps’ volume within the TEC with a flat base was significantly greater than the one under the baseless TEC at day 80 (Fig. 5b). Flaps’ maximum growth rate was enhanced within the TEC with a base (Fig. 5b and c). In the baseless TEC conditions, MRI images unveiled that the TEC appeared filled with underlying tissues (Fig. 5d). As a result, the flap tissue was compressed without any additional extension possibility (Fig. 5d). Altogether, these results indicate that a flat base is required in optimum TEC design.

### Fat flap growth under bioresorbable TEC

**Figure 6:**
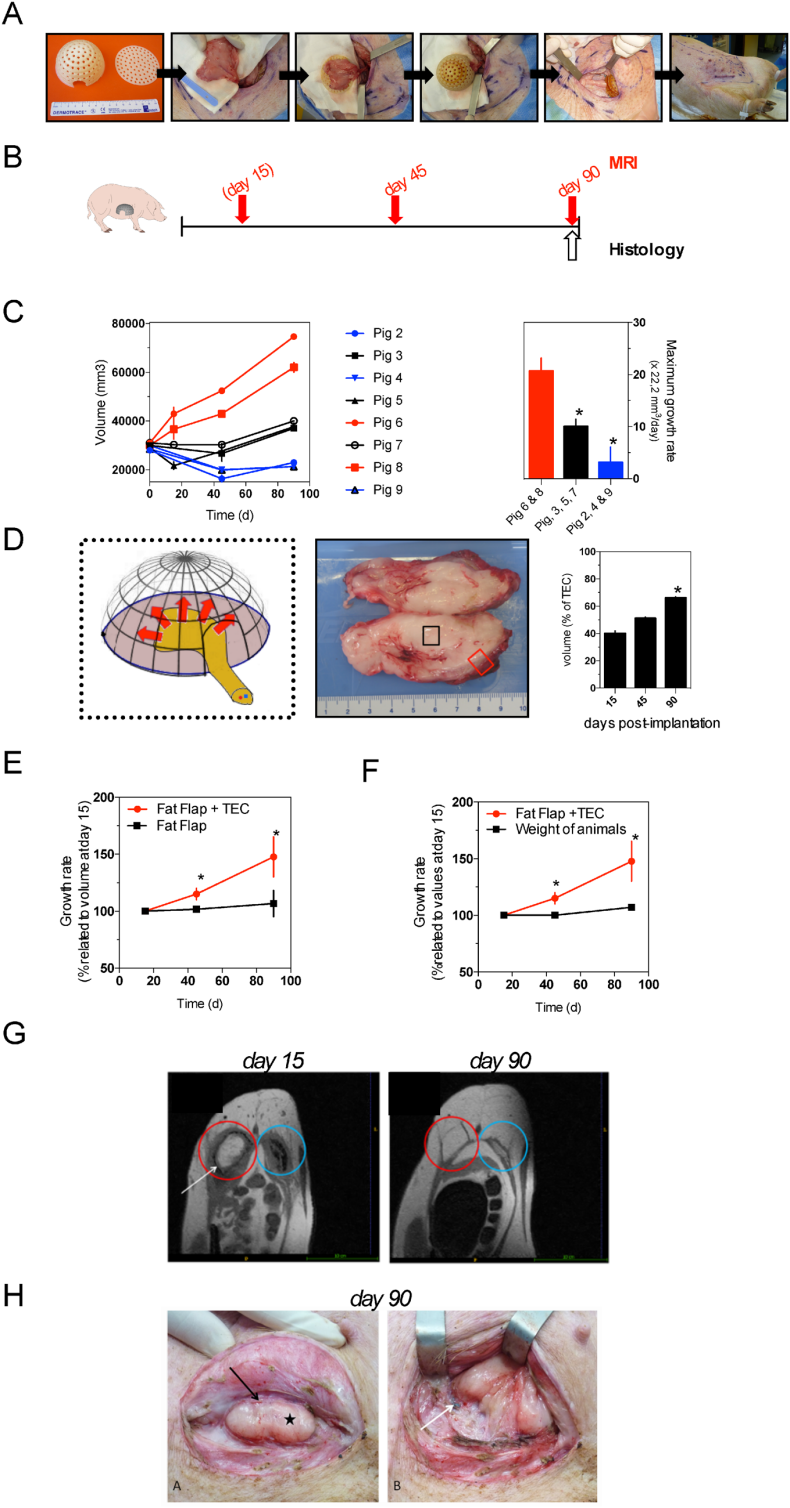
In vivo growth of fat flap under bioresorbable TEC (**a**) Surgical procedure of implanting the TEC in pig. The PGS growth chamber comprised of a flat base and a perforated dome-shaped lip. (Note the hole on the dome for the insertion of the pedicle vessels); lifting the pedicled adipose flap suture of the flap onto the base of the TEC with threads; suture of the dome on the base with threads; transferring the TEC and flap in the subcutaneous pouch and fixing on the underlying muscular aponeurosis; immediate postoperative aspect after watertight closure on 3 planes; (**b**) Experimental design: schematic depicting the follow-up of pigs implanted with bioresorbable multiperforated TEC (TEC 6 see Table 2) by MRI and histological evaluations at the indicated timepoints (MRI were performed at day 15 only for pigs 5, 6, 7, 8 (**c**) MRI quantitative analysis of in vivo growth kinetics of adipose tissue within bioresorbable TEC. Results are expressed as means ± SD in 8 pigs. Depending on growth rates we grouped pigs into 3 groups (Pigs 6 & 8, pigs 3, 5 and 7, pigs 2, 4 & 9). * p < 0.05; (**d**) Drawing of the set-up showing the pedicled adipose flap (yellow) inserted in the TEC in order to obtain adipose growth (red arrows); Gross morphology of the fat flap after insertion in the TEC; cross sections of fat tissues at day 90 after implantation. Note capsule of fibrovascular tissue at the peripheral of the TEC; Growth of adipose tissue within the bioresorbable TEC at days 15, 45 and 90 after implantation in pigs (n = 8). Results are expressed as means ± SD of percentages of the theoretical volume within the TEC * p > 0.05; (**e**) Comparison of relative growth rates of fat flap within the bioresorbable TEC and without the TEC. Results are expressed as means ± SD; (**f**) Comparison of relative growth rates of fat flap within bioresorbable TEC and weight of pigs. * p < 0.05; (**g**) MRI images in axial sections (T1 3D sequences) of fat flap within the TEC at day 15 (*left panel*) and day 90 *(right panel*). The fat flap within the TEC (red circle) is in hypersignal T1 with the TEC (white arrow, TEC visible at day 13 and not at day 83) being resorbed around it. In pig 6, an empty TEC without flap (blue circle) was used as control. Note the empty TEC without flap being resorbed then disappeared at day 83 leaving place to fibrosis; (**h**) Photograph showing a 90-day fat flap specimen (black star) within the bioresorbable chamber. Note that resorption was complete for the dome though some parts of the base had remained (white arrow). The figure was produced, in part, by using Servier Medical Art https://smart.servier.com/.

Next, we assessed whether a significant growth of adipose tissue could be obtained when the fat flap was inserted into a bioresorbable TEC (TEC 6, see Fig. 1). To do that, we kept the TEC model described above and adapted it for a larger volume (theorical volume: 114 000 mm^3^) in a porcine model (Fig. 6a and Table 2 for details). As shown in Fig. 6a, the superficial circumflex iliac adipose fat flaps (starting flap volumes around 30,000 mm^3^) were placed within the TEC in the iliac region of several pigs (Fig. 6a), then volumes of adipose tissue were calculated from MRI images over time (Fig. 6b and 6c). For the duration of the experiments, we did not observe abnormal clinical signs in the implanted pigs, which appeared healthy, and no local inflammatory reactions were detected in the skin overlying the TEC. MRI analyses of adipose tissue volumes pointed to an important heterogeneity level in tissue growth (volumes ranged from 20,000 mm^3^ to a maximum of 75,000 mm^3^; mean volume across all pigs 39,675 mm^3^ ± 19,850 at day 90 postoperative) (Fig. 6c, left panel). According to the heterogeneity of responses, we grouped pigs into three separate groups with different levels of growth rate: increased, null or decreased growth rate over time (Fig. 6c, right panel). In the high-growth group the generated adipose tissue filled the TEC volume up to 66% at day 90 postoperative (Fig. 6d). The macroscopic inspection of adipose tissues did not reveal significant inflammatory responses or the presence of necrotic tissues and conversely blood was macroscopically visible on the surface of the newformed tissue (Fig. 6d). For 3 different pigs, the fat flap was introduced into the TEC on one iliac side although on the contralateral side the fat flap alone was prepared without a TEC and served as a control. The comparison of tissue growth between fat flap alone vs. TEC clearly indicated that the presence of TEC accelerated the growth of adipose tissue over time (Fig. 6e). Furthermore, adipose tissue growth within the TEC was not simply due to the pigs’ weight gain (Fig. 6f). According to MRI images (Fig. 6g), the TEC resorption was quasi-complete at day 90 post-implantation. Likewise, during the in vivo study at 90 days, almost all TECs had resorbed with a disappearance of the dome within 3 months. A small piece of the base and the non-resorbable threads used to fix them persisted at the contact of the flap (Fig. 6h). In vivo and for all cases, a pulsatile flow was found at the section of the flap’s pedicle suggesting that flaps were all properly vascularized.

Altogether these results indicate that the bioresorbable TEC can support adipose tissue growth in vivo.

### In vivo consequences of bioresorbable TEC implantation

**Figure 7:**
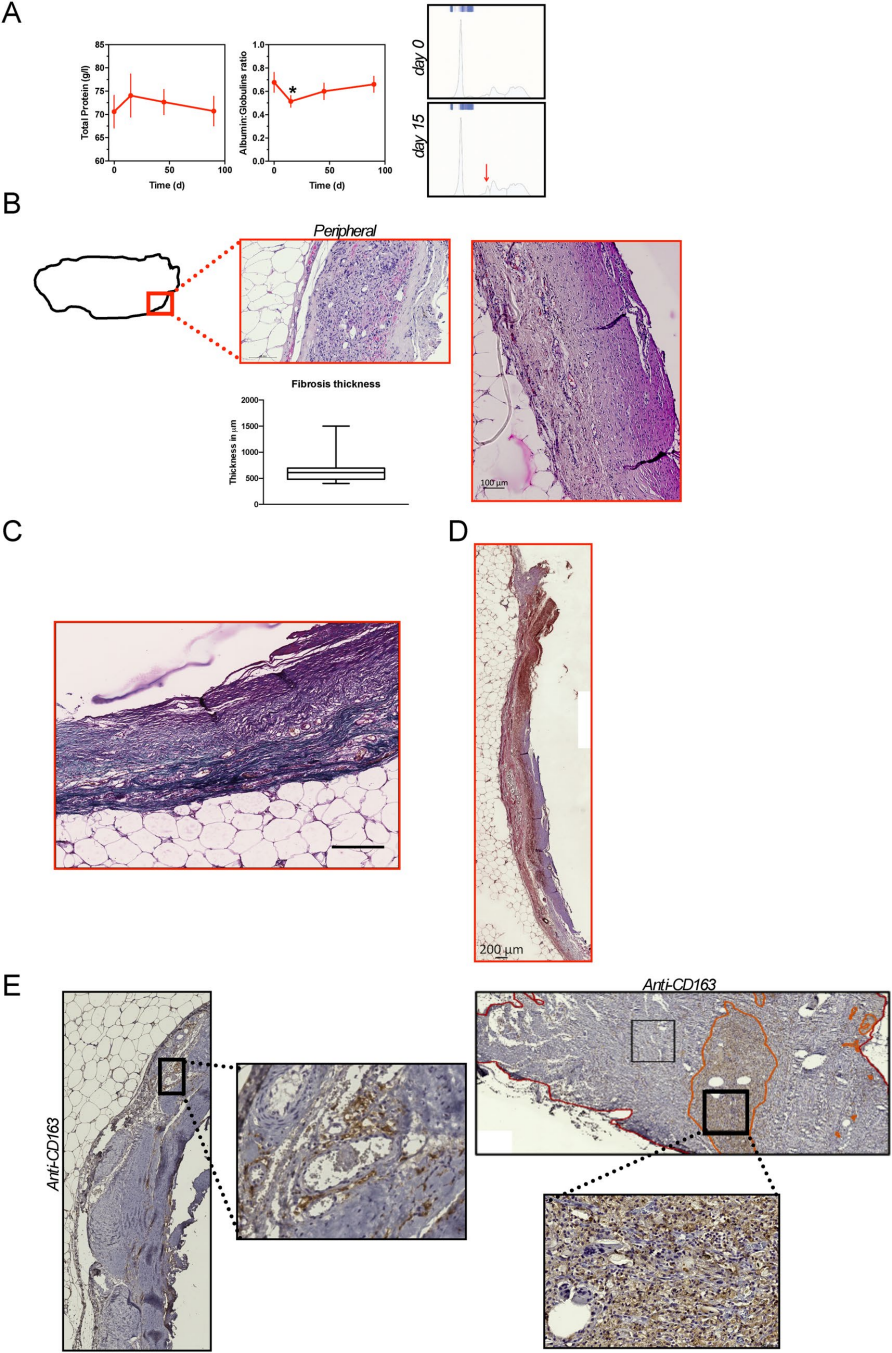
In vivo consequences of implanting the bioresorbable TEC (**a**) Evaluation over time of total serum protein and albumin:globulin ratio in pigs (n = 8) implanted within the bioresorbable TEC. Data are expressed as means ± SD. * p < 0.05 in comparison with results at day 0. Representative electrophoretic profiles of serum proteins from pigs implanted within the bioresorbable TEC at day 0 and day 15 post-implantation. The red arrow shows an alpha2 globulins peak; (**b**–**d**) Morphological evaluation of tissues harvested from the peripheral parts of the flap within the bioresorbable TEC at 90 days post-implantation; (**b**) Representative image of HE stained tissue, higher magnification view of connective tissue is shown on the *right*. Scales as marked. (*lower*) Whiskers plot (median 5–95 percentile) showing thicknesses of the fibrous capsule surrounding the adipose tissue; (**c**) Representative image of Masson’s Trichrome stained tissue; (**d**) Representative image of anti-α SMA^+^ stained cells in the connective capsule. (**e**) Representative images of anti-CD163-stained cells in the fat flap. (*left and upper*) Higher magnification views of positive representative areas in the connective tissue are also shown.

We also sought to determine the systemic and local foreign-body reactions of bioresorbable TEC implantation for 90 days (Figs. 6b and 7). Serum levels of total protein and albumin/globulin ratio were measured in pigs over time (Fig. 7a). No difference was observed in total protein serum levels over time after TEC implantation. However, we observed a significant drop in the A/G ratio at 15 days post-surgery. This drop, corresponding to an increase in the alpha2 proteins as evidenced by the electrophoresis, was transient since A/G levels remained unchanged at days 45 and 90 post-surgery (Fig. 7a). Consistently, long-term values of total protein and A/G ratio (measured at day 113 post-surgery) reversed to normal (Fig. 7a). Thus, the implantation of a bioresorbable TEC caused a transient systemic inflammatory response that normalized with time. Histomorphological analyses were performed using HE and Masson’s trichrome staining methods to evaluate local foreign-body reactions of the bioresorbable TEC. (Fig. 7b and c). Serial tissue sections at the peripheral zone of the fat flap were obtained followed by low magnification observation. In the peripheral zone of the fat flap, a thin encapsulation of the adipose tissue was observed with fibrous connective tissue capsular rim (Fig. 7b). The fibrotic capsule was also identified from Masson’s trichrome staining as a dense layer of collagen fibers aligned parallel to the surface (blue staining) with variable local density of α-SMA + myofibroblasts (Fig. 7d) and infiltration of macrophages (Fig. 7e). The infiltration of CD163^+^ macrophages was located in specific areas representing around 13.5% of the fibrous connective tissues (Fig. 7e).

### Histological study of the adipose tissue under the bioresorbable TEC

**Figure 8:**
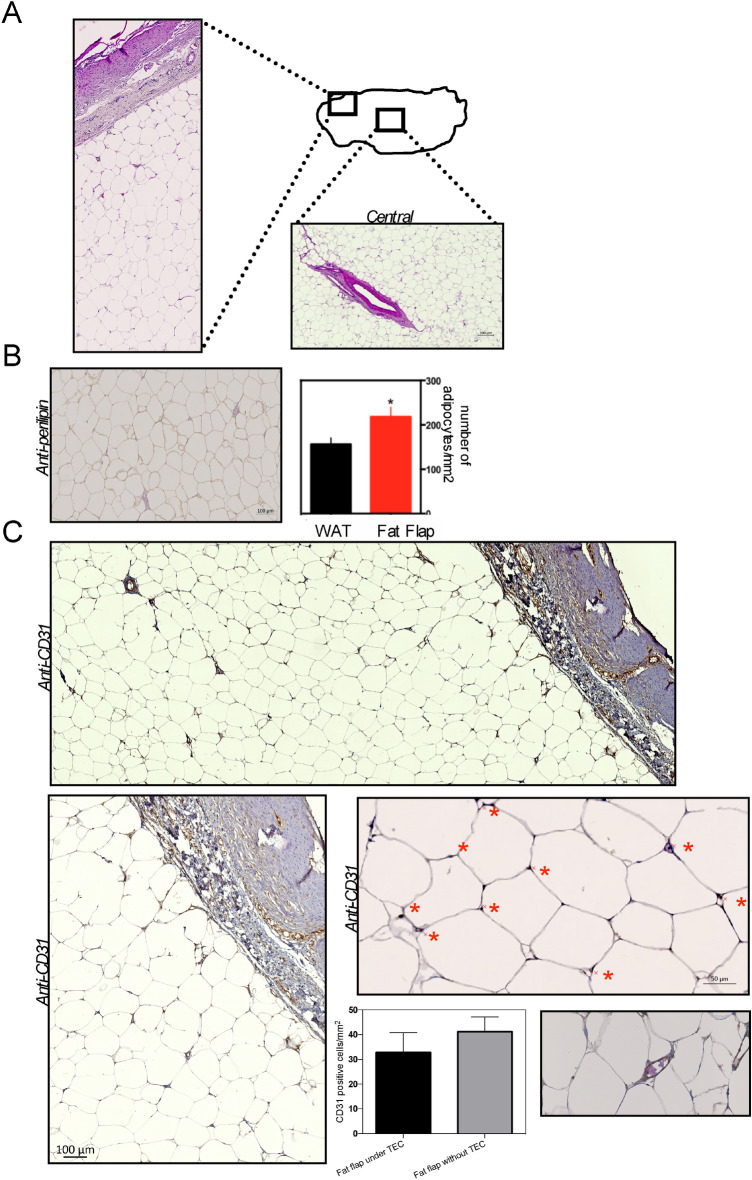
Morphological characterization of the adipose tissue within the bioresorbable TEC at day 90 post-implantation. (**a**) Representative images of HE stained adipose tissue harvested from the central and peripheral parts of the flap within the bioresorbable TEC at 90 days post-implantation; (**b**) *left* Perilipin-stained section of the adipose tissue within the bioresorbable TEC. Scale bar as indicated. (*right*) Quantification showed that the adipose tissue within the bioresorbable TEC had a higher adipocyte content than normal fat used as control (*p < 0.05); (**c**) Representative photographs of healthy, well-vascularized, adipose tissue with CD31^+^ capillaries within the bioresorbable TEC. Note also the well-vascularized connective tissue above fat tissue. Blood vessel walls are stained dark brown (red stars), and lumens with some containing red blood cells are visible at higher magnification. Comparison of the number of CD31^+^ cells per mm^2^ of fat flap within the bioresorbable TEC and without the TEC. Results are expressed as means ± SD.

Next, we performed histologic studies of adipose tissues with HE staining. After volume measurement, the adipose tissue was cut into slices to harvest distinct macroscopic areas (Fig. 8a). In the central area, the presence of blood vessels surrounded by regular adipocytes was observed (Fig. 8a). The peripheral area also consisted of mature fat tissue under the fibrous capsule at the outermost layer (Fig. 8a). Adipocytes were of normal size, with normal lobular architecture and no signs of atrophy or hypertrophy were detected. The absence of flap necrosis and inflammatory cell invasion in the adipose tissue confirmed the macroscopic data presented above. In the area between the peripheral and central areas, almost all cells were positive for perilipin, a marker of mature adipocytes (Fig. 8b). To verify if the fat flap expansion resulted from an increased number of adipocytes (hyperplasia) or size of adipocytes (hypertrophy), we analyzed the adipose cell number per mm2 of tissue (Fig. 8b). Compared to control white adipose tissue of pigs, the fat flap had more adipocytes per surface area whereas no significant increase in adipocyte size was observed, indicating that the TEC favors fat flap growth mainly by stimulating the proliferation of adipocytes (Fig. 8b). Thus, growth of the fat flap under the TEC could be attributed to adipose tissue regeneration.

The extensive tissue ingrowth was supported by well-developed blood vessels as illustrated by CD31 immunohistochemistry (Fig. 8c). Evidence of angiogenesis in fat flap under the TEC was similar to that in the group without TEC (Fig. 8c). A network of new blood vessels was noted mainly at the junction between the adipose and connective tissues extending into the inner zone of fat flap (Fig. 8c).

## Discussion

Previous studies largely demonstrated the ability of TECs to promote adipogenesis and angiogenesis (for review^1^).

In this paper, we provide suggestions for improving existing TECs. Firstly, we demonstrated that the presence of large pores (diameters 1.5–2 mm) into the dome was a crucial factor that hastened fat flap’s growth. Unlike within perforated TEC, the growth of adipose tissue under closed TECs, was inconsistent and occurred later on (starting around 100 days) (Fig. 2). These results are in agreement with previous studies evidencing the absent gowth of adipose tissue within a closed TEC even at 20 weeks^17^. In the light of macroscopic evaluations, our histological analyses of enlarged fat flaps (Fig. 3) revealed that the adipose tissue regeneration under perforated TECs consisted predominantly of mature adipocytes. In agreement, based upon HRMAS NMR spectroscopy, we provided the confirmation of the functionality of the engineered adipose tissue. Besides, histomorphometry revealed that the adipose tissue structure was normal and supported by consistent vascularization disseminated throughout the fat flap. In addition, we observed a large number of small adipocytes in peripheral areas of the fat tissue corresponding to areas of high-density neovascularization. This observation underlines the tight association of adipogenesis with angiogenesis required for optimal growth and maintenance of engineered adipose tissue. Interestingly, adipocyte progenitor cells, which are smaller than mature adipocytes^21^, reside within the perivascular region of adipose tissue. It is tempting to speculate that small adipocytes represent progenitor cells that could proliferate and differentiate into mature adipocytes to regenerate the adipose tissue. Such small progenitor adipocytes are derived from endogenous fat flap-resident stem cells and/or of blood-derived stem cells^22^. We also noticed higher neo-micro vessel density (CD31^+^ cells) in the peripheral area of the engineered tissue. i.e. *in* the regions below the TEC. This is compatible with a model in which TEC creates a gradient of ischemia spreading from the central vessel outward to the edge of the TEC^12^. Another possible explanation of the rich vascularization in peripheral areas is that angiogenic stimuli was derived not only from the pedicle of the flap but mostly from the surrounding connective tissues observed in the vicinity of the TEC (Fig. 3).

The requirement of dome permeability for adipose growth suggests that angiogenic and growth factors as along with nutrients located outside the TEC device increase the stimulation of the fat flap’s growth. Indeed, cells (mainly macrophages) surrounding the TEC express numerous cytokines and inflammatory factors promoting angiogenesis and adipogenesis. This is most likely due to macrophage production either through direct secretion or through indirect proteolytic extra-cellular matrix remodeling of pro-angiogenic factors such as vascular endothelial growth factor (VEGF), platelet derived growth factor (PDGF) or tumor necrosis factor-α (TNF-α) as well as the pro-adipogenic factor, fibroblast growth factor-2 (FGF-2)^23^. This paracrine communication was clearly demonstrated in another in vivo model (fluid drainage model), where the exudate found within the TEC also had the ability to trigger adipogenesis remotely^14^. In a TEC model for bone formation, an alternative to a perforated dome was the presence of mesh walls, which showed improved angiogenesis and tissue growth within the porous TEC^24,25^. In a silicon TEC, infiltrated macrophages switched gradually from a pro- inflammatory M1 macrophage phenotype toward a reparative (M2) macrophage phenotype during adipose tissue regeneration^26^. In several experimental models of tissue regeneration, M2 macrophages orchestrated angiogenesis, extra-cellular matrix remodeling and stem cell regulation to elicit tissue expansion^27^. Similarly, we found a significant amount of M2 macrophages in both rat and pig TEC models as revealed by the CD68 and CD163 staining, respectively. These macrophages may drive the angiogenesis and subsequent adipogenesis observed in the fat tissue.

Moreover, perforated domes may enable the formation of a functional vascular network in the fat flap linking the extrinsic vascularization (from outside the TEC) to the intrinsic vascularization (in the fat flap)^16,17^. Thus, pore size less than 400 µm in porous scaffold hinders blood vessels penetration^28^. In a rat model, bioresorbable scaffold with pore sizes larger than 1 mm promoted angiogenic invasion from surrounding tissues^29^. In the presentstudy, we conceived perforated TEC with pore size of at least 1.5 mm, which could be compatible with optimal vascular infiltration into the TEC as observed in the periphery of engineered tissue. It was also hypothesized TEC perforation could influence morphological and biological characteristics of the surrounding connective tissue around the adipose flap resulting in altered mechanical tensions on the fat flap, thus accelerating adipogenesis^17^.

Secondly, we demonstrated that the presence of a flat base under the adipose flap was an important determinant for adipose growth within the TEC device. As suggested by MRI images, the presence of a flat base provides protection against compression and strain of underlying tissues allowing the harmonious and continuous development of adipose tissue within the TEC. However, it is also possible that the flat base influences the fat flap cells that are on it by mechanical transduction pathways activating the proliferation of adipocytes^30^. In line with this, mesenchymal stem cells cultured on a matrix support mimicking the stiffness of natural adipose tissue do upregulate adipogenic markers^31^.

Currently, most existing TECs are permanent and have the inherent limitation of leaving a foreign material within the body. Permanent TECs could lead to the development of chronic adverse host responses, such as chronic inflammation with prominent alteration of surrounding tissues leading to an abundant fibrotic capsule^10,16,17^. The thick fibrotic capsule may occupy a large space under the TEC and prevents fat tissue from developing. The thick capsule is also responsible for cutting off external communication, thereby resulting in nutrient and oxygen shortage as well as reducing angiogenic infiltration^16^ . In addition, when tissue growth is achieved, permanent TEC must be explanted requiring an additional surgical procedure for removal. Given these drawbacks, there has been growing interest in developing temporary bioresorbable TECs. Bioresorbable TECs also represent an attractive solution by avoiding risks and costs linked to secondary surgery for TEC removal.

The biodegradable polymer PLGA was used for TEC applications but it induced major foreign body reactions, due to possible decreases in pH, that might contribute to the abundant production of fibrotic capsule representing more than 30% of the fat flap^10,17^. Conversely, in our system, the use of a PGS bioresorbable TEC did not induce systemic chronic inflammation and the space under the TEC was almost totally filled with newly formed adipose tissue. Although we observed the presence of a fibrotic capsule around the engineering adipose tissue, its thickness was limited and did not impede neo-vascularization and fat flap growth (Figs. 7 and 8). Indeed, our observations (Fig. 8) indicate that bioresorbable TEC allows the enlargement of adipose flap through efficient adipogenesis (hyperplasia) sustained by neo-vascularization (CD31^+^ cells). This result is compatible with the observation that a mild inflammation is required to promote the neovascularization and adipogenesis processes whereas the development of excessive chronic inflammation is deleterious to fat growth^8^. The challenge of temporary TECs is that the bioresorbable materials need to provide volume and shape stability to allow for adipose tissue growth within the TEC followed by a rapid resorption to avoid chronic fibrosis. Recently, a preliminary study reported for the first time the safe use of a bioresorbable mold to shape a fat flap in rats^32^. However, due to the experimental model, the final adipose tissue volume was low^32^, tenfold lower than the one reported in our study. Despite the heterogeneity of the final volume obtained across all pigs, the mean volume of tissue created was around 10,000 mm^3^ corresponding to the difference between the initial volume and the volume at day 90. Thus, we demonstrated that bio-resorbable TECs are able to promote tissue integration to achieve significant adipose tissue volume up to a maximal final volume of 75,000 mm^3^, similar to the one already obtained with a non-resorbable polycarbonate TEC in a pig model^10^.

According to the manufacturer, the Purasorb PGS Polyglycolide used in 3D printing of the TEC progressively loses its strength after 6 months and completely disappears in 12 months. However, we observed in the in vivo experiments a quicker degradation of the bioresorbable TEC domes that had completely disappeared within 3 months. In order for the polymer to not be absorbed too rapidly, it is important for TEC domes to remain mechanically stable (uncollapsible) during tissue development to maintain an “empty space” for tissue growth and resist to contraction forces of the surrounding tissues. The absorption speed of Purasorb PGS could have been affected by altered polymers after 3D printing or the higher body temperature of pigs vs. humans. As a probable result of the premature TEC absorption, we observed that the theoretical volume of the chamber was not completely filled by the adipose tissue at day 90 (Fig. 6). Our lab is working to design bioresorbable TECs with few FDA-approved polymers that were well characterized^33^, and that could degrade after 90 days allowing for complete tissue regeneration.

Novel findings of our study are also that PGS is a medical-grade nontoxic biomaterial compatible with 3D printing processes making it possible to manufacture 3D printed customized TECs. Indeed, size and shape of the tissues to be regenerated may require personalization to best fit the anatomy of each individual and therefore TECs may need to be customized. In fact, 3D printing medical scaffolds were tailored for breast reconstruction using medical imaging techniques demonstrating the potential interest in the medical field^34^.

TECs exhibit a great potential for tissue regeneration but several challenges still persist before translating into clinical applications. As previously reported^10,15^, it is difficult to predict the final volume within the TEC. Here, we observed a large heterogeneity in the growth rate of adipose tissue within the bioresorbable TEC (Fig. 6). This could be due to the quality of the fat flap vascularization inserted into the TECs. The existence of vascular pedicle spasms, occurrence of vascular torsion or obstruction during fat flap dissection could give inconsistent results in term of adipose tissue regeneration. The heterogeneity of the growth tissue could be compensated by adding further improvements to the TEC design such as the perforated dome and flat base, the simultaneous use of external devices^35^, and/or preconditioning strategies^11^.

Our study had some limitations that warrant attention. The first one being the small sample size and nonoptimal animal model sites, especially for pigs, with a continuous compression that could have altered the TEC structure. The second one resides in the short follow up of the generated tissues. An additional follow up period could enable further analysis of the long term stability of the engineered tissue as previously reported^10^.

To conclude, our findings can be summarized as follows: (1) the design of TEC requires the presence of multiple pores within the dome with a pore size of at least 1.5 mm to allow optimal tissue growth; (2) the design of TEC requires the presence of a flat base to protect the growing adipose flap against compression and strain of underlying tissues; and (3) 3D-printed bioresorbable TECs allow the growth of large volume vascularized, viable mature adipose tissue without inducing a prominent inflammatory response.

Further studies are needed before the translation of these approaches is applied to clinical practice. The results presented here may help to achieve this goal.

## Methods

### Tissue engineering chambers (TEC)

Two polymers were used to design the different types of TEC (see Table 2) according to their resorption kinetics. Regarding its long degradation time (> 24 months in humans), polylactic acid (PLA) PLA Chromatik (Dagoma, France) was used as filament to design a long-term bioresorbable TEC. Short-term bioresorbable TEC prototypes were made from medical-grade poly[1,4-dioxane-2,5-dione] : polyglycolic acid (PGA) PURASORB PGS (Corbion Purac, The Netherlands). PURASORB PGS is a semi-crystalline polymer and was extruded into 1.75 mm filament for 3D printing. Its biodegradation time ranges from 6 to 12 months according to the manufacturer. TEC n° 1,2,3 and 6 prototypes were modeled with Blender software, before being printed by Fused Deposition Modeling (FDM) on Ultimaker2 3D printer (Ultimaker, The Netherlands). Alternatively, TEC n°3 and 4 were designed on Solidworks (Waltham, MA) and fabricated using a DiscoEasy200 3D printer (3D Dagoma, France). These TECs were kept in a waterproof bag away from humidity and at + 4 °C until animal implantation.

### Materials characterization

PLA filament and PURASORB PGS pellets thermal properties (characteristics are shown in Table 1) were analysed using a TA instruments DSC 2,920 Differential Scanning Calorimetry (DSC). Samples weighing 5–10 mg were placed in aluminium pans and were heated from − 20 to 240 °C with a heating and cooling rate of 10 °C/min. The melting temperature (Tm) were determined at the maximum temperature at the endothermal peak and the glass transition temperature (Tg) was taken at the Midpoint ISO. Thermogravimetric analyses (TGA) were performed using a TA 2050 Instruments at 10 °C/min from 20 to 700 °C. The degradation temperature was defined as the temperature at which the sample loses 5% of its initial mass (Td5%).

### Animal models

Animal experiments were performed under GMP conditions in accordance of standard protocols of the Department of laboratory animal facility (University of Lille, School of medicine). These protocols were conducted under the regulation of the Review Board of the Ethics committee (University of Lille, School of medicine). All experiments described were submitted to and approved by the regional Ethics committee (CEEA 75 North region of France) with the agreement of the French ministry of higher education and research (N°2015083019275331). A total of 8 female Wistar rats weighing 270 ± 50 g were maintained under a 12-h day/ night cycle in pathogen–free conditions and fed normal chow and water ad libitum. 8 female adult minipigs with initial weights ranging from 57.5 to 90.5 kg, had enough subcutaneous fat to proceed with subcutaneous adipose flaps to implant “human-size” prostheses in subcutaneous pouch. Animals were weighed before each surgery and before each MRI. Study outcomes (Histology, MRI) were assessed in a blinded manner.

### TEC insertion in rats

Rats (n = 12) were anesthetized with inhaled isoflurane (Abbot) at 5 l/min for induction and at 2 l/min for maintenance, in combination with oxygen. TECs were implanted subcutaneously in the dorsolumbar region of Wistar rats following the recently suggested refinements^13^. Animals were shaved on the back and then put on a heated plated at 37 °C in ventral decubitus. The implantation site was cleaned with betadine and a sterile field is set up over the animal. A 4 cm incision was performed, just under the lowest rib of each animal. After incision, subcutaneous conjunctive tissues were dissected in order to separate the skin from the muscle plan, up the thigh to create a subcutaneous pocket. Care was taken to protect the different vessels as well as dorsal muscles.

A subcutaneous fat flap was then dissected by separating the subcutaneous fat of the skin without harming the vascular pedicle and cut out to fit inside the 3D printed mold. After dissecting the pedicle fat flap, the fat flap was spread and sutured with non-absorbable sutures (Prolene 4–0, Ethicon) to the base of the chamber. The volume of fat flap was determined using 3D-printed molds [original mold volume = 180 mm^3^ for rat 2 and rat 3 (Fig. 2); 130 mm^3^ for rats 4 to 7 (Fig. 2) and 740 mm^3^ for rats implanted with TEC4 or TEC5 (Fig. 5)]. The dome of the chamber was then sutured to the base, ensuring that the door was positioned over the pedicle with no risk of harming the pedicle. The chamber was then sutured to the dorsal muscle with non-absorbable sutures for stability.

The subcutaneous pocket was then closed with a continuous suture between the subcutaneous plan and the dorsal fascia. Subsequently, wound closure was performed with a continuous suture using absorbable suture (Monocryl 3–0, Ethicon). The wound was then cleaned with betadine and the animal returned to a clean cage.

After awakening, each animal received a subcutaneous injection of 0.2 mg/kg of Buprenorphine (Buprecare, Axience).

All TEC insertions were unilateral excepted when the base’s role was tested (Fig. 5). For this particular protocol, each rat (n = 3 for TEC4 and n = 3 for TEC5) received one TEC consisting of a dome-shaped device set on a circular base on the left side and one TEC consisting of a baseless dome-shaped device on the right side.

Pigs received a premedication via an intramuscular injection consisting of Ketamine (10 mg/kg) and Xylazine (2 mg/kg) 30 min before the procedure. Pigs were intubated and ventilated under general anesthesia with Isoflurane, furthermore a postoperative antibiotic prophylaxis was also put into place. TEC were disinfected by a wash in 10% pure topical Betadine solution, for 30 min. For technical simplicity and animal comfort, fat flaps based on the ipsilateral superficial circumflex iliac vessels in the groin were dissected as previously mentioned^10^. Initially, the fat flap volume was estimated using the volume displacement method and was generally around 30,000 mm3 (mean 29,625 ± 1,200 mm^3^). The fat flaps were inserted within the TEC before subcutaneous implantation (Fig. 6a). For 3 pigs, the contralateral fat flaps were also harvested without TEC implantation. Otherwise, contralateral sides were used as controls.

### Magnetic resonance imaging

The volume of the flaps was measured using magnetic resonance imaging (MRI) when indicated. MRI was performed using the imaging systems operating at 0.2 T (Hitachi, Airis Mate) for pigs or at 7 T (Bruker, Biospec, Ettlingen, Germany) for rats. Animal sedation was maintained during the entire imaging procedure.

T1, T2, T1 3D and T1-weighted images before and after gadolinium injection were acquired. Slice thickness was 5 mm or 2 mm for 3D sequences. Post-processing of MRI images (including three-dimensional manipulation and volume measurements) was performed by three independent operators using manually drawn, serial ROIs in ITK-SNAP 3.2 software or OsiriX imaging software (64-bit version 3.8.1, Pixmeo©, Geneva, Switzerland).

### ^1^H-NMR method

Each sample (20 to 26 mg of fat tissue generated in the TEC and of subcutaneous fat harvested from the back of the same animal in the area contralateral to the flap as control) was stored at − 80 °C until analysis. Tissue was suspended in a solution of D_2_O (99.9%) with TMSP-d_4_ before being loaded on the NMR sample rotor (4 mm ZrO_2_). After loading the fat tissue, a 50 µL insert was placed in the sample holder to stabilize the sample and to provide the balance for the rotor. The preparation of NMR samples was done rapidly after take out of the freezer to avoid sample degradation. The TMSP-d_4_ is used as an external chemical shift reference (δ = 0 ppm).^1^H-NMR experiments were performed at 4 °C using a Bruker NEO 500 MHz liquid-state NMR spectrometer, with a 4 mm HR-MAS probe. The probe-head was pre-cooled to 4 °C before loading the sample into the instrument. The sample spinning rate was controlled in the range of 3,000 Hz (± 1 Hz). Spectra were acquired with suppression of water signal. The pre-saturation of the water signal was achieved with a zgpr sequence before the pulse. The 90° pulse length was calibrated and adjusted based on each sample. The number of transients was 80. A repetition time of 5 s and a spectral width of 12 kHz were used. The data were processed using software TOPSPIN 4. A line broading (0.3 Hz) apodization was applied to all FIDs before Fourier transformation. For resonance assignment and confirmation, a two-dimensional ^1^H J-coupled correlated spectroscopy (COSY; standard bruker pulse sequence) was used to determine J-couple correlations of resonance. COSY was collected with 10 kHz spectral width in both dimension and a 1.5 s relaxation delay; 10 transients were averaged for each of the 128 increments in D1, corresponding to a total acquisition time of 35 min. Two-dimensional spectral data were analyzed on the instrument, using zero filing to a 1 k × 1 k matrix and weighted with a shifted square sine bell function and 1 Hz exponential (D1 and D2) line broading, followed by Fourrier transformation.

### Tissue assessment for histomorphometry and immunohistochemistry

At the time of sacrifice, macroscopic evaluation of the fat flaps was performed before the histological assessments. Flaps retrieved were measured, weighed, photographed and the volume was evaluated. Then, all tissue harvested from the chambers were immediately fixed with 10% Paraformaldehyde overnight. Tissues were then embedded in paraffin and serially cross-sectioned perpendicular to the axis of the pedicle vascular bundle. 10 µm-thick histological specimens were prepared at each section and routinely stained with hematoxylin and eosin. Masson's trichrome staining was performed to detect the collagen deposition in tissue. The total collagen content was reported as a percentage of the counter staining divided by the total tissue area of the section using ImageJ software (National Institutes of Health, Bethesda, MD, America)^36^. The thickness of connective tissues was evaluated randomly with ImageJ software as previously detailed^29^). To evaluate the presence of vessels and the quality of adipocytes in the implantation site, slides were stained for CD31 antibody (rabbit anti mouse CD31, 1:50 dilution, BD Biosciences, MA) or for perilipin antibody (1 µg/ml ab126639 Abcam), respectively. Adipocyte Tools plugin (Montpellier RIO imaging platform, Montpellier, France) developed on ImageJ (National Institutes of Health, MA, USA) was used for automated computing of cell size and cell number distribution. We also evaluated the macrophage distribution in rat and minipig specimens with anti-CD68 (mouse monoclonal anti-CD68 1:500 Abcam: ab31630) and anti-CD163 (mouse monoclonal anti-CD163 1:200 NB110-40,686 Novus) immunohistochemical staining, respectively. The antibody against α-smooth muscle actin (anti α-SMA, monoclonal antibody 0.5ug/ml A5228-200UL Sigma) was used to detect the presence of myofibroblasts in the fat flap. For each staining, isotype controls were used as negative controls. To detect the primary antibodies, the Vectastain ABC kit, (Vector Laboratories, Burlingame, CA, USA) was used following standard procedures. Slides were counterstained with hematoxylin. All slides were assessed after scanning with Axioscan Z.1 (Zeiss, Germany) and analyzed with Zen 2012 blue edition software (Zeiss). All measurements were performed in blinded manner by different examiners on randomly chosen sections from each group.

### Biological measurements

Local fluids and serum were collected from rats and pigs in each group and were then stored at − 80 °C until analysis. Proteins in serum and fluids were analyzed by automated capillary electrophoresis system (Capillarys 2 Sebia, Norcross, GA).

### Statistical analysis

Results are presented as mean ± SD. Statistics were done with a dedicated software: Graphpad Prism 8 (Graphpad Software™, Inc., San Diego, USA). We used two-way analysis of variance to compare groups at all time points, and non-parametric *t* tests to compare two groups at a single time point. The level of significance was set at * p ≤ 0.05.
